# The Medical Aspect of Iowa

**Published:** 1867-03

**Authors:** A. G. Field

**Affiliations:** Des Moines, Iowa


*The Medical Aspect of Iowa.* By A. G. Field, M.D., Des Moines, Iowa.

By nature, Iowa has been liberally endowed. Its rolling prairies stretching out in graceful undulations, like the waves of the ocean; its beautiful rivers flowing along their deep, well-washed channels; and its perpetual breeze, scented in summer by the fragrant breath of an endless variety of wild flowers, are all characteristic. So, also, are the fine development, the health, the energy, and the vivacity of her citizens.

On the map, Iowa extends from 40° 20′ North, to 43° 30′, embracing about 3 degrees in the middle of the North Temperate Zone, and the climate is not less salubrious than is thus indicated by its position. The mean temperature in summer, is about 70° Fahrenheit, and that of winter, about 20°. Rain is almost always accompanied by a sensible diminution in the temperature of the atmosphere.

Iowa is, emphatically, a prairie state, not, however, abounding in the broad and monotonous prairie plains of her sister on the east, but in prairies of less area, which approach a level on the high lands, but become more undulating and rolling in the low lands and near the water courses. The highest prairie is, therefore, often wet and cold, and less fertile than that which is more undulating. About four-fifths of the area of the State is prairie land, which, in summer, is clothed with the most luxuriant growth of vegetation; and if the waves of the ocean, sparkling in the sunbeams, *seem* to be clothed in sparkling gems, those of dry land *are, indeed*, in the more varied beauty of the endless variety of wild flowers which adorn them.

Sloughs are an interesting feature of a prairie country. In the absence of more desirable water, they furnish a supply for very many new settlers, and, although they are mere pools of standing water, they do not become stagnant, nor possessed of malarious properties. This is due, in part, to their depth, as well as to their protection from the heat of the sun by the long grass and their overhanging margins, from the tenacity of the sod, permitting, in many instances, large sub-sod excavations to be formed by the water. Slough water is much more palatable than its name indicates to a "down-easter." It has a sweetish, loamy taste, is quite free from the elements of any kind of decomposition, and, so far as I know, is not considered unhealthy.

In Iowa, there are few, if any, large tracts of heavy-timbered land to breathe out their poisonous exhalations into the surrounding atmosphere. The bottom lands are the timbered lands. The streams are all skirted with a belt of timber that, on either side, extends from a few rods to a mile or two, or more, in width.

The atmosphere is rich in oxygen, and from the large excess of vegetable life that feeds upon it, we may, perhaps legitimately infer that it is poor in carbon. I do not know that ozone has been detected in it, but have reason to believe that it exists. In my own person, respiration, in health, is from 15 to 20 per cent faster in the atmosphere of New York City than in that of Iowa, and many claim to have experienced a buoyancy and elasticity of spirit, upon coming here, that was entirely unknown to them in the Eastern States.

The almost constant prairie breeze augments the severity of the climate in winter, and renders the heat of summer less oppressive. The prevailing winds in summer are from the south and south-west; and in winter from the north and north-west.

Southerly winds are usually bland and grateful, but seldom continue long without bringing a storm, often resulting, no doubt, from the condensation of a highly rarified atmosphere. The north-westers of winter are of much severity, blowing, as they do, parallel with the course of the streams, and being chilled by the great snow regions of the mountains. Northerly winds are, next to easterly, most rare, and, when they do occur, are of very short duration. Easterly winds occur less frequently than those from any other source, and are also of short duration. They are not cold, but bring a humid atmosphere with them from the great lakes, and are more chilly and intolerable even than those from the north. Westerly winds, in summer time, are always refreshing, and they are more constant than those from any other source. All are extremely variable, and with every change in the direction of the wind, there is generally a corresponding change in the temperature of the atmosphere.

On the whole, the climate of Iowa, although subject to sudden changes, and, to some extent also, to extremes of heat and cold, is not only favorable to general health, but certainly exercises a curative influence over the scrofulous and tubercular diathesis. The constant wind exerts, also a mitigating influence over malarious influences where they exist, and endemics are almost entirely averted.

In enumerating some of the more characteristic causes of disease in Iowa and other prairie States, no allusion will be made to many of those which are confined to the cities and large, well-built towns. In these, the citizens enjoy the conveniences and luxuries of their former eastern homes, and are subject to many of the causes of disease that they were in them. But "far out upon the prairie," whither the adventurous young farmers go to *make* themselves homes, are to be found those especial influences and causes of disease to which we would allude. Here, the infirmities of old age and of chronic diseases seldom go. A very large proportion of new settlers are young and middle-aged, have good constitutions, and the "snap" of energy. Society is made up from the more healthy and ambitious people of communities in other states and countries, each bringing the peculiar manners, customs, and mode of living of his nationality. Diversity in these things, results in diversity in the causes and complications of disease. The physician must study his patient as well as his disease.

The pneumonia which attacks a robust man who has labored all through the heat of summer and the cold of winter, is quite a different disease, considered therapeutically, from that which attacks his friend of sedentary habits just from his warm house in an eastern home. And so, too, is the dysentery of the child just from its native home in Georgia, with relaxed fibre and impaired nervous force, quite a different disease, therapeutically considered, from the dysentery of another child, whose athletic frame and smutty face speak of the privations and neglect and simple mode of living of its native new country home. In newly settled countries, common privations in living as well as in the pursuit of accustomed avocations, are met in various ways; thus originating new influences and causes of disease, as diversified almost as are the individuals themselves. And besides these, the great differences in the habits and predispositions of individuals from different States and countries, demand of the physician the most attentive observation and careful study. With this allusion to some of the causes of disease which relate to the subjects of it, we will proceed to notice some of those which more properly belong to a new prairie country. Preëminent among these may be mentioned the dwelling-houses.

Many new settlers live for a year or more in small, open, half-finished houses, of but a single room, in which to live, sleep, cook, eat, and wash, with no out-houses, and with slough water for family use, which must be carried from one to three hundred yards. That the exposures incident should result in inflammatory and other diseases, is obvious; and then, in addition to the other uses of the already over-crowded little room, it also becomes a sick room. O, how the heart has ached a hundred times in such a sick room. The only wonder is that any in it should live, to say nothing of the sick.

Other especial causes of disease result either directly or indirectly from the great excess in the vegetable form of life, affecting in obvious ways the constitution of the atmosphere. The animal subsists by extracting the oxygen in respiration, and exhales carbon. The vegetable subsists upon carbon and exhales oxygen, chiefly; the one form of life thereby constituting the atmosphere for the subsistence of the other, and each being indispensible to the well-being of the other. The wind is constantly equalizing the proportions of the atmospheric elements; but a great excess of either form of life in a given quantity of atmosphere obviously operates to diminish the proportion of the peculiar elements upon which it subsists, as well as to increase the proportion of the elements evolved by it, however great the diffusibility of these gasses.

That this conclusion is not altogether hypothetical may derive some support from the experience of very many who in a prairie atmosphere have realized its exhilarating effect. A new energy and motion seem often given to the whole man, mentally, as well as physically, and even old people bending under the weight of many years, have been known to regain much of the vivacity and activity of early life upon removing to a prairie state, and to continue to enjoy for years a degree of health as gratifying as it has been surprising. In the absence of any demonstration by analysis, have we not some ground for the conclusion that the atmosphere of Iowa is rich in oxygen? But how far, or under what peculiar circumstances this fact may relate to disease as well as to health, we will not now attempt to determine. But if it is true that the existence of this excess of the vegetable form of life exerts an influence upon the constitution of the atmosphere, it is equally true that its sudden destruction, or the decomposition therefrom resulting, must produce other and equally important changes in it to affect the animal economy.

An autumnal frost blights in a single night the grassy domain of an hundred prairies, and reverses the whole order of nature's phenomena. The laws of chemical change triumph over those of vital change, and the living, breathing plant, becomes at once a mass of incipient decomposition, oozing at every pore the putrid gasses of decay. But comparatively, only a very small proportion of prairie grass is permitted to undergo slow decomposition. Nature provides a more speedy and fortunate means of resolving it back into the original elements, in the great prairie fires that sweep, and sometimes with thundering fury, over hundreds of square miles in a single day, and continue their devastating march for days and even weeks without abatement. The result is that large quantities of smoke, and vapor, and carbonic acid gas, are suddenly liberated into the atmosphere to pervert and disease the functions of animal life. Coincident with the occurrence of frost and of prairie fires, begin also the more serious cases of autumnal diseases, dysentery, cholera-morbus, and intermittent and remittent fevers. These fevers generally evince a marked tendency to the typhoid form of disease if not arrested early, although idiopathic typhoid fever rarely if ever occurs. We are accustomed to call the typhoid form of disease typhoid fever, simply because it is convenient to do so. Other causes no doubt are largely concerned in the production of these diseases, even in Iowa, but because of their universality we make no allusion to them in this article. Another means by which a large amount of prairie grass is caused to undergo decomposition every year, is by "prairie breaking." The sod, which is a dense mat of vegetable matter and earth, from three to six inches in thickness, as well as the luxuriant growth of the season, is caused to undergo decomposition in summer time, and as a consequence a malarious emanation is evolved. All over our young State, the energetic farmers are every year making large inroads upon the "raw prairie," with the prairie plow. That this decomposition is a fruitful source of malaria, is evident in the fact that families living near recent breakings, during a summer and autumn, seldom escape malarious disease. I think I have never known a family to escape, who lived near by, and north or east of, a large breaking during a summer. In Iowa, malarious poisoning, from whatever source originating, does not occur in the malignant or intensified form that it does in some malarious districts in other States; but wherever there is heat, moisture, and decomposing organic matter, we may expect to have malaria in greater or less amount, and if Dr. Salisbury's doctrine be true, perhaps we may regard even it as an example of nature's provident goodness, exhibited in an effort to again tie up into organization the elements which have just been set at liberty, and which are so detrimental to the perfect well-being of the animal economy. The water courses, as a source of such emanations in Iowa, are not sufficiently dissimilar from those of other States to deserve much consideration. They have, generally, high, abrupt banks, and are skirted with belts of timber to drink up, to a great extent, the elements of their miasmatic emanations. But bottom lands, subject to overflow, as well as swales and pools of standing water along the course of, and near, the streams, are a very common source of miasmatic poisoning, and families exposed to either of these in summer time seldom escape.

The causes of inflammation, to some of which allusion has already been made, are, perhaps, more numerous and more potent for evil than are those of any other class of diseases. They occur most abundantly in winter and early spring, but from the very nature of the conditions upon which they depend, they are in perpetual operation. These are, firstly, a predisposition by the plethora and the habits of a large majority of the people. Secondly, exposures incident to small, unfinished, and insufficient houses; and, thirdly, the sudden reduction of the temperature of the atmosphere, by the frequent changes in the course of the wind. Of this class of diseases, the following may be mentioned as most common, in the order in which they are enumerated, *viz.*:—Catarrh, pneumonia and pleuro-pneumonia, croup, conjunctivitis, erysipelas, meningitis, gastro enteritis, etc. Like malarious diseases, some of these also have a marked tendency to assume the typhoid form early, and although decidedly sthenic in the onset, the antiphlogistic treatment is required with exceeding caution in very many instances, while others will not bear it at all.

Epidemics have nothing peculiarly characteristic in them. What may, I believe, be said of all epidemics, is especially true of those which have prevailed in Iowa, *viz.*:—that the first cases are attended with the greatest fatality, with a decreasing ratio in proportion to the length of time which they prevail. The diseases which have most frequently assumed this form are, diphtheria, dysentery, erysipelas, and scarlatina. Diphtheria has prevailed with much fatality, especially with small children, in the last five years. Cerebro-spinal meningitis, in some localities, has also been attended with much, and even greater, fatality, in proportion to the number of cases, than any other disease of which I have any knowledge. The first epidemic of it occurred in the winter of 1863–4, since which it has occasionally manifested itself in some portions of the State.

There is another predisposing cause of disease in Iowa, about which nothing, so far as I know, has been said, and yet which should not be overlooked. It is the want of a properly acidulated diet in spring time and summer, the result of which is, probably, an abnormal constitution of the blood. I do not pretend to know just what this abnormal condition consists of, but it has been clearly and repeatedly demonstrated that the use of acids removes it. We know that the blood does sometimes possess an abnormal alkaline reäction, and that most acids, when ingested, enter the circulation as such, and combining with the salts of sōda and other bases, restore the blood to its normal constitution. We know, also, that the capillary circulation is enfeebled, and that there are other morbid phenomena which attend these cases, in hypinosis, and again the acids, especially the mineral, are restorative by increasing the fibrine and albumen, and, thereby, also the circulation in the capillaries.

Whether either of these conditions of the blood, *viz.*:—its abnormal alkalinity, or its want of a due proportion of fibrine and albumen, is the condition under consideration, we do not venture to assert; but that the change of seasons brings about a demand on the part of the bodies of both man and animals, for a change in the constitution or quality of food, and that such demand is, most happily, responded to on the part of nature by the varied provisions of her storehouse, is beyond question.

Fruits are a necessity as well as a luxury, and nature supplies them just when they are most needed, but not to the prairie pioneer, who, having ventured beyond the limits of her provident fruit garden, as well as those of his fellows, must plant, and prune, and wait for——his own.

